# Diagnosis and Staging of Metabolic Dysfunction-Associated Steatotic Liver Disease Using Biomarker-Directed Aptamer Panels

**DOI:** 10.3390/biom15020255

**Published:** 2025-02-10

**Authors:** Mikkel B. Kjær, Asger G. Jørgensen, Søren Fjelstrup, Daniel M. Dupont, Claus Bus, Peter L. Eriksen, Karen L. Thomsen, Jeyanthini Risikesan, Søren Nielsen, Charlotte W. Wernberg, Mette M. Lauridsen, Elisabetta Bugianesi, Chiara Rosso, Henning Grønbæk, Jørgen Kjems

**Affiliations:** 1 Department of Hepatology and Gastroenterology, Aarhus University Hospital, Palle Juul-Jensens Boulevard 99, 8200 Aarhus N, Denmark; mikj@clin.au.dk (M.B.K.); ple@clin.au.dk (P.L.E.); karethom@rm.dk (K.L.T.); 2 Department of Clinical Medicine, Aarhus University Hospital, Palle Juul-Jensens Boulevard 99, 8200 Aarhus N, Denmark; 3 Steno Diabetes Center Aarhus, Aarhus University Hospital, Palle Juul-Jensens Boulevard 11, 8200 Aarhus N, Denmark; yeyakana@rm.dk (J.R.); soeren.nielsen@clin.au.dk (S.N.); 4 Interdisciplinary Nanoscience Centre (iNANO), Department of Molecular Biology and Genetics, Aarhus University, Gustav Wieds Vej 14, 8000 Aarhus C, Denmark; asgergivskov@inano.au.dk (A.G.J.); soeren.fjelstrup@inano.au.dk (S.F.); dmd@inano.au.dk (D.M.D.); cb@inano.au.dk (C.B.); 5 Department of Gastroenterology and Hepatology, University Hospital of Southern Denmark, 6700 Esbjerg, Denmark; charlotte.wilhelmina.wernberg@rsyd.dk (C.W.W.); mette.enok.munk.lauridsen@rsyd.dk (M.M.L.); 6 ATLAS Centre for Functional Genomics, University of Southern Denmark, 5230 Odense, Denmark; 7 Department of Medical Sciences, University of Turin, Via Verdi 8, 10124 Torino, Italy; elisabetta.bugianesi@unito.it (E.B.); chiara.rosso@unito.it (C.R.)

**Keywords:** biomarker, aptamer, metabolic dysfunction-associated steatotic liver disease, metabolic dysfunction-associated steatohepatitis

## Abstract

Metabolic dysfunction-associated steatotic liver disease (MASLD) affects one-third of adults globally. Despite efforts to develop non-invasive diagnostic tools, liver biopsy remains the gold standard for diagnosing metabolic dysfunction-associated steatohepatitis (MASH) and assessing fibrosis. This study investigated RNA aptamer panels, selected using APTASHAPE technology, for non-invasive MASLD diagnosis and fibrosis stratification. Aptamer panels were selected in a cohort of individuals with MASLD (development cohort, *n =* 77) and tested in separate cohorts: one with MASLD (test cohort, *n =* 57) and one assessed for bariatric surgery (*bariatric cohort*, *n =* 62). A panel distinguishing MASLD without steatohepatitis from MASH accurately stratified individuals in the development cohort (AUC = 0.83) but failed in the test and bariatric cohorts. It did, however, distinguish healthy controls from individuals with MASLD, achieving an AUC of 0.72 in the test cohort. A panel for fibrosis stratification differentiated F0 from F3–4 fibrosis in the development cohort (AUC = 0.68) but not in other cohorts. Mass spectrometry identified five plasma proteins as potential targets of the discriminative aptamers, with complement factor H suggested as a novel MASLD biomarker. In conclusion, APTASHAPE shows promise as a non-invasive tool for diagnosing and staging MASLD and identifying associated plasma biomarkers.

## 1. Introduction

MASLD is a leading cause of liver-related morbidity and mortality [1] and is estimated to affect 30% of adults [2,3]. Furthermore, MASLD is the most rapidly growing contributor to liver-related morbidity and mortality globally [4]. MASLD covers a histological and clinical disease continuum from simple hepatic steatosis to the progressive phenotype of metabolic dysfunction-associated steatohepatitis (MASH) with or without hepatic fibrosis [5]. MASLD displays considerable interindividual heterogeneity, and recent studies have underlined this by identifying that a subgroup of individuals with MASLD progresses rapidly from simple steatosis to inflammation and advanced fibrosis [6,7]. Furthermore, the natural history of MASLD includes temporal fluctuations in disease severity and fibrosis degree, with disease progression and regression well described in the literature [7,8,9]. The nomenclature for non-alcoholic fatty liver disease (NAFLD) has recently been proposed to be changed to MASLD according to a consensus statement by multiple liver disease societies [10]. Initial assessment of concordance between MASLD and NAFLD diagnosis shows an almost complete overlap between these diagnoses [11,12]. Thus, this work will adhere to the novel MASLD terminology.

The diagnosis of MASLD relies on the presence of hepatic steatosis and one of five cardiometabolic risk factors [10,13,14]. Meanwhile, the diagnosis of MASH requires the simultaneous presence of steatosis, lobular inflammation, and hepatocyte ballooning in a liver biopsy. This procedure not only struggles with sampling variability [15], but its invasive nature makes it unpleasant for the patients, costly, and, in many instances, inadequate for the longitudinal evaluation of MASLD severity in a clinical setting or the design of interventional trials. Despite comprehensive research on biomarkers, composite scores, and imaging techniques for diagnosing MASH, liver biopsy remains the gold standard [16,17,18,19]. Thus, the increasing global burden and the considerable clinical heterogeneity of this disease warrant the development of efficient non-invasive diagnostic tools, particularly those able to identify individuals with a high risk of progression to advanced disease and poor prognosis as well as those able to monitor the effect of interventions in MASLD.

We recently described a novel method, APTASHAPE, to evaluate and identify biomarkers in plasma when applied to bladder cancer patients [20]. The APTASHAPE technology involves a selection step of a large pool of chemically modified RNA oligonucleotides, termed aptamers, capable of specific binding to any protein epitope present in the human plasma proteome. The number of bound aptamers directly reflects protein content and/or modifications between samples, thus enabling the identification of biomarkers based on aptamer binding profiles [21].

In the present study, our primary aim was to investigate the utility of the APTASHAPE technology to select biomarker-directed aptamer panels capable of non-invasively detecting MASH and fibrosis stages in individuals with MASLD. These aptamer panels were validated in a separate cohort of individuals with MASLD and a cohort of individuals assessed for eligibility for bariatric surgery. Our secondary aim was to identify putative protein targets for the selected discriminative aptamers by mass spectrometry (MS).

## 2. Methods

### Study Design and Population

To conduct the APTASHAPE experiments, 255 plasma samples from individuals with MASLD, severe obesity, or healthy comparators were collected. The samples were included via three established cohorts: an Italian cohort of individuals with MASLD [22], a Danish cohort of individuals with MASLD and healthy controls [23,24], and a Danish cohort of individuals enrolled when screened for eligibility for bariatric surgery [25].

A total of 77 individuals with MASLD from Turin, Italy, were included to identify aptamers discriminating MASLD without steatohepatitis and MASH or fibrosis stages. The cohort was collected from 2010–2012 with core data published previously [22]. This cohort will be referred to as the development cohort. Two cohorts with a collective 57 individuals (47 with MASLD and 10 healthy controls) collected at Aarhus University Hospital between 2015 and 2021 were combined and used to test the performance of the selected aptamer panels in a MASLD population. This combined cohort is referred to as the test cohort. Lastly, a cohort of 62 individuals from the ongoing PROMETHEUS study [25] collected between June 2018 and June 2022 at the University Hospital of Southern Denmark, Esbjerg, Denmark, was used to test the accuracy of the selected aptamer panels in a cohort of bariatric individuals. This cohort, referred to as the bariatric cohort, contained histological data from baseline and end-of-study (EOS) liver biopsies collected 18 months post-baseline biopsy. These samples are referred to as the baseline and EOS samples. Fibrosis staging in the included cohorts was conducted by Kleiner fibrosis scoring [26]. The histological diagnosis of MASH was based on the fatty liver inhibition of progression (FLIP) algorithm [27] in all cohorts except the bariatric cohort, where a NAFLD activity score (NAS) ≥4 was used [26]. Samples in all cohorts were grouped according to the presence of steatohepatitis (healthy, MAFLD without steatohepatitis, and MASH) and fibrosis stage (F0, F1–F2, and F3–F4). The study was approved by the Central Denmark Region Committee on Biomedical Research Ethics (no. 1-10-72-48-20, no. 1-10-72-213-18, no. 1-10-72-283-18, no. 1-10-72-344-18, no. 1-16-02-322-15, and no. 1-52-81-302-20) and the Danish Data Protection Agency (no. 2022-522-0500).

## 3. APTASHAPE Analysis

The APTASHAPE analysis of plasma samples was performed using an established pipeline described previously with minor modifications [20]. A naïve pool of 10^15^ random RNA transcripts was synthesized and selected for binding to pooled plasma proteins from 30 individuals with MASLD from the development cohort. The protein-binding RNA transcripts (aptamers) were captured on magnetic beads and enriched through four iterative rounds of SELEX, i.e., positive selection. Matrix-binding aptamers were partitioned using BSA-coated magnetic beads (counter-selection). A machine learning approach was used to select separate aptamer panels for stratifying the presence or absence of steatohepatitis or the fibrosis stage based on histological scoring in the development cohort, as previously described [20].

### 3.1. Statistical Analysis

Baseline characteristics presented in Table 1 are presented as medians with interquartile ranges for continuous variables and as numbers with percentages for categorical data. Between-group comparisons in boxplots were calculated using the Wilcoxon rank test. Aptamer discovery and aptamer panel selection were conducted using an in-house R (4.3.3) analysis pipeline [20]. First, the aptamers were ordered according to rank in the entire dataset: the aptamer with the highest number of counts was assigned rank 1, the aptamer with the second highest number of counts was assigned rank 2, and so forth. Next, the total number of counts of aptamers was plotted in boxplots by grouping variables (disease severity, sex, age, etc.) to identify outliers and investigate if these outliers were systematically present in a subset of samples. Secondly, the number of zeros for each aptamer was plotted against rank for quick interpretation of missing values. Zero values pose challenges for normalization methods, as division by zero is undefined. Furthermore, abundant sequences containing zeros across many samples will be characterized by a low signal-to-noise ratio. Thus, the number of aptamers included in the data analysis was based on this plot, and a cut-off was defined when a steep increase in zeroes appeared. Based on this methodology, the 500 most abundant aptamers were used for panel selection (Supplementary S1). Only aptamers with a fold change in regression coefficient between any two groups of interest of 0.25 and a Benjamini-Hochberg corrected *p*-value of 0.05 were considered discriminative between stages of disease (MASLD without steatohepatitis vs. MASH) or fibrosis (F0 vs. F3–4). The fold change cut-off of 0.25 was chosen based on the variation in protein abundances of the included samples in the study. In addition, preliminary studies showed that a fold change of 0.25 was sufficient to identify aptamers with discriminatory abilities. The Benjamini–Hochberg method was utilized to account for multiple hypothesis testing.

The performance of the selected aptamer panels and established scores for non-invasive assessment of MASLD disease severity and fibrosis in the included cohorts was assessed by the area under the receiver operating characteristic curve (AUC) values with 95% confidence intervals (95% CIs) calculated using the DeLong method from the R package (version 4.3.3) pROC (version 1.18.5) [28]. AUC values for the selected aptamer panels were calculated based on the discriminative aptamers’ principal component 1 (PC1) values. Non-invasive measures of disease severity included in this study were cytokeratin 18 (CK18) and soluble CD163 (sCD163). Non-invasive measures of fibrosis degree included the Fibrosis-4 index (FIB-4) and FibroScan. AUC values were qualified as acceptable (AUCs 0.70–0.80), excellent (AUCs 0.80–0.90), and outstanding (AUCs > 0.90) [29].

### 3.2. Mass Spectrometry

Aptamers selected for protein target analysis were selected by identifying discriminative aptamers in the development cohort. Aptamers that distinguished between histological components of MASLD (MASLD without steatohepatitis vs. MASH and F1–2 vs. F3–4 fibrosis) or parameters associated with MASLD (LDL concentration, sCD163 concentration, or the presence/absence of metabolic syndrome) were considered for target identification by MS. The median values of LDL and sCD163 in the development cohort were used as cut-offs. Subsequently, aptamers were evaluated for sequence similarity. Aptamers sharing extended sequence motifs (conserved regions) were considered members of the same aptamer family, most likely binding to the same protein epitope. Thus, the most abundant aptamer from each family was selected for target identification. Methods for affinity purification of proteins, MS experimental setup, and data analysis can be found in Supplementary S2.

## 4. Results

### 4.1. Study Cohort Characteristics

The clinical characteristics of the cohorts are listed in Table 1 and summarized here. The development and test cohorts were representative of the general MASLD population with elevated BMI, total cholesterol, and low-density lipoprotein (LDL). The *development cohort* differed from the *test cohort* by being younger and more severely affected by MASLD, with a higher proportion of individuals with MASH and fibrosis. This was supported by non-invasive measures of liver disease being consistently more elevated in the development cohort compared to the test cohort (liver stiffness, FIB-4, and AST/ALT ratio). Furthermore, the proportion of individuals with type 2 diabetes mellitus (T2D) was higher in the development cohort. The bariatric cohort had a higher BMI than the development cohort, with a median BMI > 35 in both baseline and EOS samples. The proportion of individuals with T2D was slightly larger in the bariatric cohort compared to the development cohort. Most of the individuals in the bariatric cohort had no MASLD or MASLD without steatohepatitis, and the disease severity in the EOS samples was the lowest due to regression of disease severity in the individuals who underwent bariatric surgery. The individuals in the bariatric cohort generally had F0-F2 fibrosis, with F3 and F4 only presenting in four (baseline samples) and five individuals (EOS samples).

### 4.2. Aptamer Panel Selection

**Figure 1:**
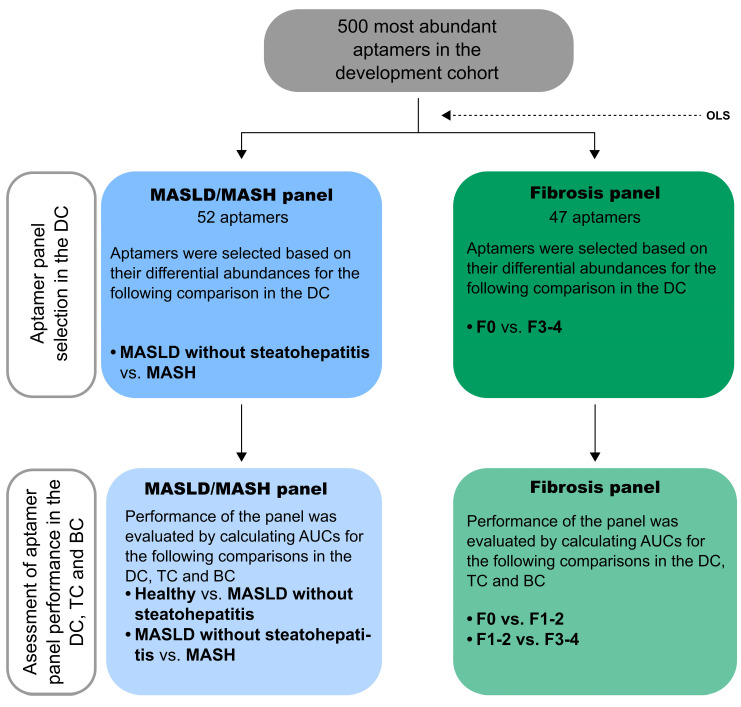
A schematic of the workflow conducted in this study to select the discriminative aptamer panels and assess their performance. The 500 most abundant aptamers in the development cohort (DC) were included, and ordinary least squares (OLS) regression analysis was performed to identify aptamers with differential abundances between groups of interest. The groups used to identify discriminative aptamers are shown in the upper row (dark blue and dark green boxes). The groups compared to assess the performance of the selected aptamer panels in the DC, test cohort (TC), and bariatric cohort (BC) are shown in the bottom row (light blue and light green boxes). Abbreviations: BC, bariatric cohort; DC, development cohort; MASH, metabolic dysfunction-associated steatotic liver disease; MASLD, metabolic dysfunction-associated steatotic liver disease; OLS, ordinary least squares; TC, test cohort.

The APTASHAPE analysis aimed to establish panels of aptamers with differential binding between individuals with MASLD without steatohepatitis vs. MASH and/or with different degrees of fibrosis. This distinction is warranted because the resolution of MASH is a common endpoint in interventional trials in MASLD and can currently only be assessed through repeat liver biopsies [30]. Furthermore, MASH has been linked to an increased fibrosis progression rate [8]. The different degrees of fibrosis were grouped into F0, F1–F2, and F3–F4 because individuals with advanced fibrosis (F3 and F4) have a higher risk of decompensation events, hepatocellular carcinoma (HCC) development, and overall mortality than those with lower fibrosis stages [31,32]. Furthermore, current standard non-invasive tests for fibrosis staging, such as FIB-4 [33], aim to rule out advanced fibrosis. The workflow for selecting and validating aptamer panels in this study is outlined in Figure 1.

### 4.3. Aptamer Panel to Distinguish MASLD with and Without MASH

**Figure 2:**
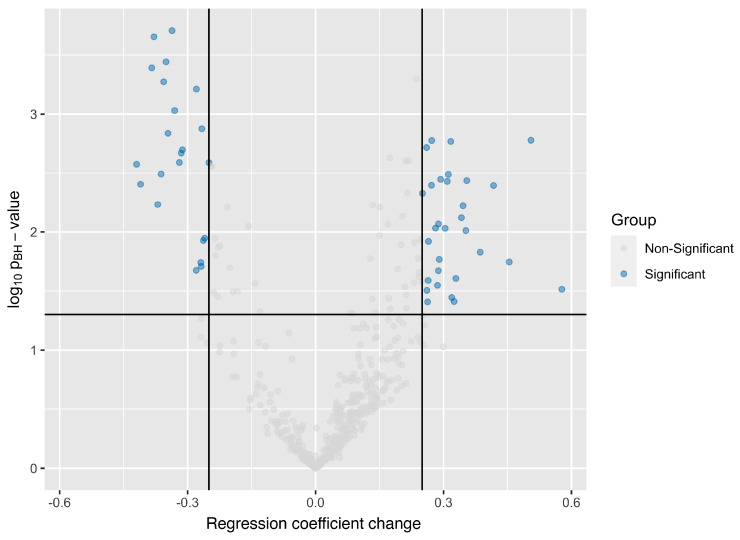
Volcano plot showing *p*-values of aptamers and their fold changes in Ordinary Least Squares (OLS) regression analysis in the development cohort for the panel to distinguish MASLD without steatohepatitis from MASH. The 500 most abundant aptamers in the aptamer pool were selected for all 77 samples and were tested for statistical significance for each aptamer. A change in regression coefficient of 0.25 (black vertical lines) and a Benjamini–Hochberg adjusted *p*-value of 0.05 (black horizontal line) were considered statistically significant for all comparisons. A comparison between patients with MASLD without steatohepatitis and patients with MASH identified 52 significant aptamers; 22 of these were down-regulated in MASH samples, while 30 aptamers were up-regulated. Abbreviations: MASH, metabolic dysfunction-associated steatohepatitis; MASH, metabolic dysfunction-associated steatotic liver disease.

**Figure 3:**
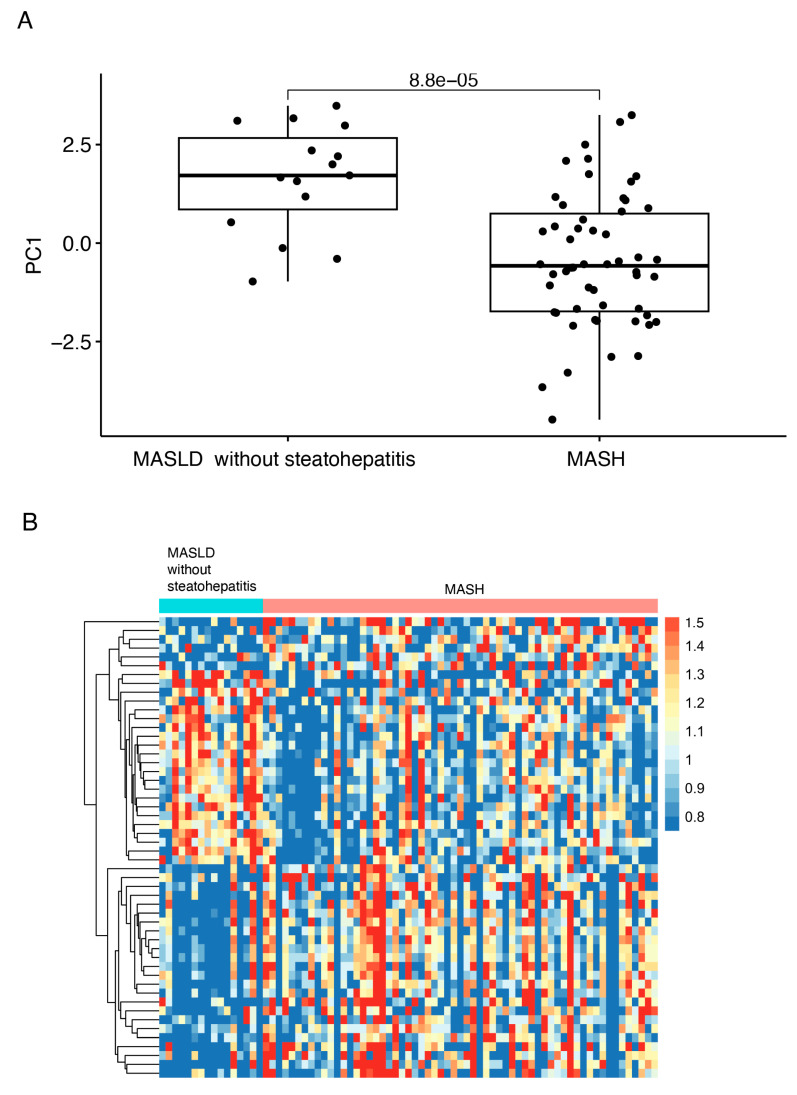
Plots of the panel to distinguish MASLD without steatohepatitis and MASH in the development cohort. (**A**) Boxplot of PC1 values of the samples in the development cohort showing statistically significant differences between MASLD without steatohepatitis and MASH groups. *p*-values were calculated using the Wilcoxon rank test, and comparisons between groups with *p*-values < 0.05 were considered statistically significant. (**B**) Heatmap of the relative abundances of the 52 aptamers capable of distinguishing MASLD without steatohepatitis from MASH in the development cohort. Abbreviations: MASH, metabolic dysfunction-associated steatohepatitis; MASH, metabolic dysfunction-associated steatotic liver disease; PC1, principal component 1.

First, we investigated the ability of the 500 most abundant aptamers to differentiate individuals with MASLD without steatohepatitis from MASH. In total, 52 aptamers had differential abundances between samples from individuals with MASLD without steatohepatitis and MASH (Figure 2) and were selected for the discriminative aptamer panel. In the development cohort, the panel discriminated between MASLD without steatohepatitis and MASH (Figure 3) with an excellent AUC of 0.83 for this comparison. Selecting an aptamer panel to distinguish between healthy and MASLD was not possible, as there were no healthy individuals in the development cohort. In the test cohort, the AUC for discrimination between healthy and MASLD without steatohepatitis using the aptamer panel was 0.72, while it declined to 0.52 for the distinction between individuals with MASLD without steatohepatitis and individuals with MASH. Similarly, in the bariatric cohort, the AUC values for the distinction between healthy individuals and individuals with MASLD without steatohepatitis was 0.60 (baseline samples) and 0.80 (EOS samples), while the AUC values for the distinction between MASLD without steatohepatitis and MASH were 0.47 (baseline samples) and 0.49 (EOS samples). AUC values with 95% CIs for the aptamer panel to distinguish MASLD without steatohepatitis and MASH are summarized in Table 2.

Thus, our selected aptamer panel has the potential to identify individuals with MASH, although we were unable to reproduce this finding in the test cohort. This is of particular interest as there is no current biomarker for this purpose. Furthermore, the aptamer panel could discriminate between healthy individuals and individuals with MASLD without steatohepatitis.

### 4.4. Aptamer Panel to Distinguish Fibrosis Stages

**Figure 4:**
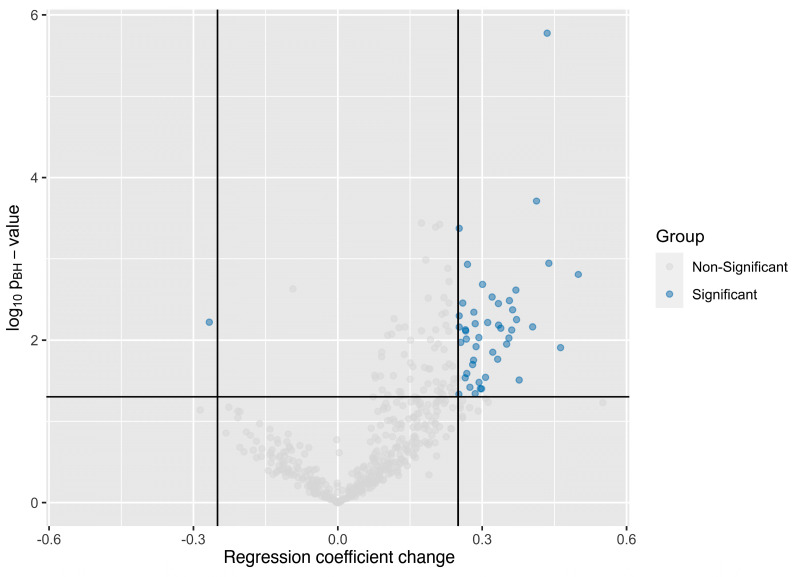
Volcano plot showing *p*-values of aptamers and their fold changes in ordinary least squares (OLS) regression analysis in the development cohort for the panel to distinguish F0 from F3–4. The 500 most abundant aptamers in the aptamer pool were selected for all 77 samples and were tested for statistical significance for each aptamer. A change in regression coefficient of 0.25 (black vertical lines) and a Benjamini–Hochberg adjusted *p*-value of 0.05 (black horizontal line) were considered statistically significant for all comparisons. A total of 47 aptamers were found to be significantly down- or up-regulated in the comparison between patients with F0 and F3–4. One of these was down-regulated in F3–4 samples, while 46 aptamers were up-regulated in F3–4 compared to F0.

**Figure 5:**
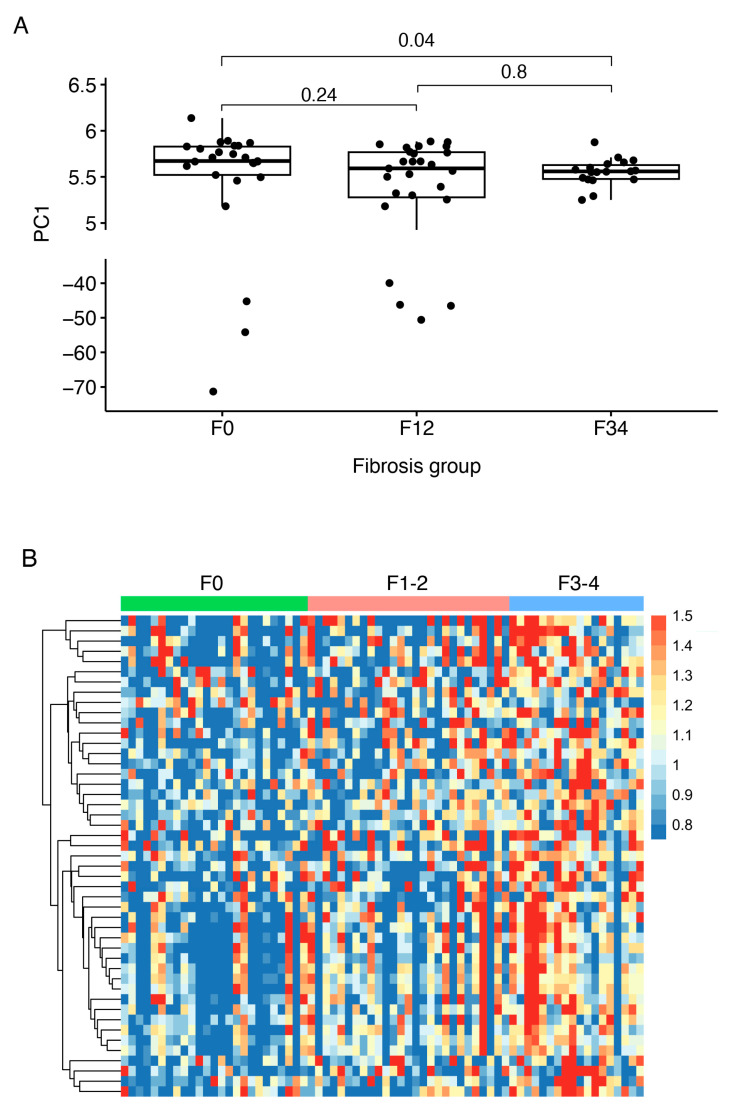
Plots of the panel to distinguish F0 from F3–4 in the development cohort. (**A**) Boxplot of PC1 values of the samples in the development cohort showing statistically significant differences between F0 and F3–4 fibrosis groups. *p*-values were calculated using the Wilcoxon rank test, and comparisons between groups with *p*-values < 0.05 were considered statistically significant. (**B**) Heatmap of the relative abundances of the 47 aptamers in the fibrosis model grouped according to fibrosis groups in the development cohort. Abbreviations: PC1, principal component 1.

Next, we tested the ability of the 500 most abundant aptamers to predict fibrosis stages in individuals with MASLD. In total, 47 aptamers showed differential abundances between samples from individuals with F0 and F3–4 and were selected for the discriminative aptamer panel (Figure 4). In the development cohort, the panel discriminated between F0 and F3–4 (Figure 5), with an AUC of 0.68 (95% CI: 0.51–0.85). The AUC for F0 vs. F1–2 was 0.60 (95% CI: 0.44–0.75); for F1–2 vs. F3–4, it was 0.52 (95% CI: 0.35–0.70).

In the test cohort, the panel showed an AUC of 0.54 for the discrimination between F0 and F1–2. Testing the panel’s ability to distinguish between F1–2 and F3–4 was not possible because no individuals in the test cohort had fibrosis stage 3 or 4. In the bariatric cohort, the panel showed AUC values of 0.69 (baseline samples) and 0.52 (EOS samples) for the discrimination between F0 and F1–2. AUC values with 95% CIs for the fibrosis panel are summarized in Table 3. In conclusion, our aptamer panel has below-acceptable performance to distinguish fibrosis stages.

### 4.5. Aptamer Target Identification

Finally, we investigated the performance of APTASHAPE as a biomarker discovery platform by identifying the putative protein targets of aptamers. In an attempt to identify a large number of unique markers that reflect the complexity of MASLD, we selected aptamers that can distinguish various aspects of MASLD for protein target analysis. In total, 28 aptamers were chosen for protein target analysis.. Of the 28 aptamers initially selected for protein target analysis, five (aptamers 8, 10, 13, 26, and 28) were also included in the aptamer panel to distinguish MASLD without steatohepatitis from MASH. A total of 49 of the included 52 aptamers in this panel contained one or more nucleotide sequence motifs used to identify aptamer families. Similarly, 4 of the 47 aptamers included in the aptamer panel to distinguish F0 from F3–4 were identical to aptamers chosen for protein target analysis (aptamers 8, 14, 17, and 26), and 43 of the included 47 aptamers contained one or more of the sequence motifs used to identify aptamer families. Thus, the majority of the aptamers selected for the panels to distinguish MASLD without steatohepatitis from MASH and fibrosis stages are suspected to belong to the same aptamer families as the aptamers selected for MS analysis and potentially bind the same target proteins. The aptamers were synthesized individually, attached to magnetic beads, and used to affinity purify their target proteins from plasma (see Supplementary S2). Putative protein targets identified by MS are listed in Table 4.

**Figure 6:**
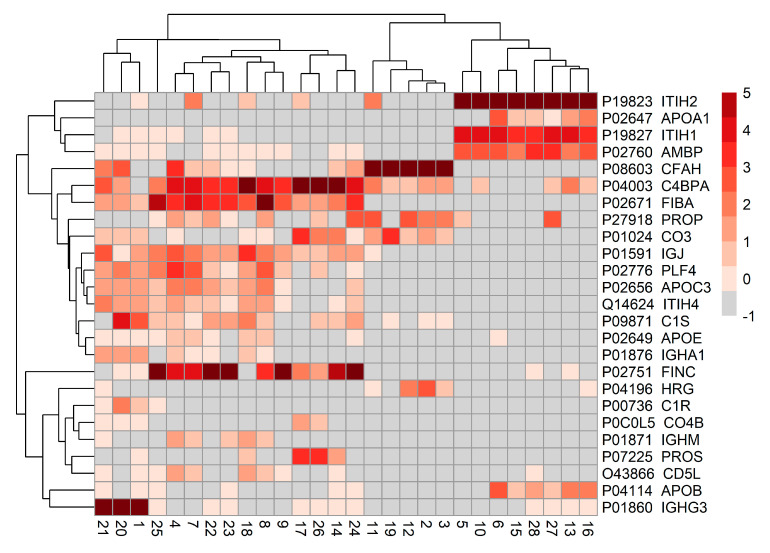
Heatmap of the relative abundance of identified proteins based on mass spectrometry of proteins co-purified with the 28 aptamers selected for protein target analysis (MS panel). The percentage of total signal intensity was calculated for each identified protein in each sample. The mean intensity was calculated for each triplicate, and the mean percentage was log2 transformed. Abbreviations of protein names and the Uniprot proteome identifiers are displayed on the right-hand side of the figure.

The MS analysis identified five main proteins in the plasma samples to be the most probable targets of the 28 discriminative aptamers selected for protein target analysis: Complement Factor H (CFH), C4b-binding protein alpha chain (C4BP), fibronectin (FN), immunoglobulin heavy constant gamma 3 (IGHG3), and inter-alpha-trypsin inhibitor heavy chain H2 (ITIH2) (Figure 6). Five aptamers had CFH as the most probable target, three aptamers had C4BP, seven aptamers had FN, three aptamers had IGHG3, and eight aptamers had ITIH2. Two aptamers had comparable amounts of FN and C4BP bound.

## 5. Discussion

In this study, we investigated the performance of the APTASHAPE technology to discriminate between individuals with different degrees of MASLD. Our main finding was that a selected aptamer panel distinguished individuals with MASLD without steatohepatitis from individuals with MASH with excellent performance in the development cohort of individuals with MASLD. Correspondingly, we identified an aptamer panel that accurately distinguished between individuals with MASLD with F0 and F3–4 in the development cohort. MS analysis identified five proteins as probable targets for the discriminative aptamers investigated.

The selected panels performed well in the development cohort, while this was not the case in the test and bariatric cohorts. One aspect potentially explaining the low performance of the aptamer panels in the test and bariatric cohorts is the differences in demographic, biochemical, and clinical parameters between cohorts. Individuals in the development cohort showed more advanced disease than the test cohort, with 79% of individuals in the development cohort having MASH and representation of all degrees of fibrosis. In comparison, less than half (42%) of the individuals in the test cohort had MASH, and none had F3 or F4 fibrosis. Furthermore, no healthy individuals were included in the development cohort, while the test and bariatric cohorts included individuals with and without MASLD. This is a limitation of the study, as the lack of healthy individuals in the development cohort may have created a bias towards aptamer panels primarily differentiating advanced disease stages. However, the aptamers included in the panel to distinguish MASLD without steatohepatitis from MASH showed the best discrimination between healthy individuals and individuals without steatohepatitis in the test and bariatric cohort, suggesting that this potential bias had only minor implications on our results.

We evaluated the performance of aptamer panels for monitoring regression in the bariatric cohort, which had data from pre- and post-intervention (bariatric surgery) liver biopsies. It is worth noting that the inclusion criteria in this cohort differed from those in the development and test cohorts, as this cohort included bariatric patients with and without MASLD. However, excluding healthy individuals when calculating the AUC for the comparison between F0, F1–2, and F3–4 did not substantially alter the performance of the developed aptamer panel to distinguish fibrosis stages. Finally, only four individuals in the baseline samples and five individuals in the EOS samples had F3–4, rendering the interpretation of AUC values for this group uncertain. The abovementioned differences between cohorts could explain the failure to reproduce the discriminative ability of the aptamer panels in the test and bariatric cohorts. Furthermore, the moderate number of samples in the individual cohorts limits the generalizability of the results, warranting studies of aptamer panels in larger MASLD cohorts.

Finally, we tested the performance of established measurements for MASLD disease severity: sCD163 and CK18 levels to discriminate between individuals with MASLD without steatohepatitis and MASH and FIB-4 and FibroScan to rule out advanced fibrosis. sCD163 showed an AUC of 0.82 for discriminating individuals with MASLD without steatohepatitis and MASH in the development cohort, comparable to the selected aptamer panels’ AUC of 0.83. Similarly, CK18 showed an AUC of 0.79 in the development cohort to distinguish MASLD without steatohepatitis and MASH, which is in line with the pooled AUC of 0.70–0.87 to diagnose MASH found in a meta-analysis [34]. However, in the test cohort, sCD163 had an AUC of 0.44, while the aptamer panel had an AUC of 0.52. We observed a similar pattern when assessing FIB-4 as a measurement to rule out F3–4. A meta-analysis showed that FIB-4 had an AUC of 0.80 for ruling out advanced fibrosis in individuals with MASLD [35]. In the development cohort, FIB-4 showed an AUC of 0.61 for the comparison between individuals with F0–2 and F3–4, thus underperforming in this cohort compared to the general MASLD population [35,36]. Liver stiffness measurements by FibroScan showed AUCs of 0.78 in the development cohort, 0.78 in the baseline samples, and 0.84 in the EOS samples. We speculate that the unknown factors responsible for the lacking performance of the established scores utilizing blood-based measurements for MASLD disease severity could equally have affected the performance of our aptamer panels.

We performed MS analysis to identify the probable protein targets of discriminative aptamers in MASLD. The analysis led to recognition of five main proteins as the most probable targets of the 28 discriminative aptamers selected for MS. The target proteins identified were CFH, C4BP, FN, IGHG3, and ITIH2. However, as the aptamer target purification was performed under native physiological conditions in plasma, we likely pulled down complexes containing multiple proteins. In accordance with this, we observed multiple proteins in the MS analysis.

ITIH2 was the most probable target of eight aptamers capable of discrimination between individuals with MASLD without steatohepatitis and MASH. This protein is constitutively produced in the liver as part of a larger ITIH family, widely associated with inflammation and fibrosis [37], MASLD, and T2D [38]. Furthermore, ITIH2 is differentially expressed in high-density lipoprotein (HDL) proteome between healthy individuals and individuals with MASLD without steatohepatitis [39]. One study in low-density lipoprotein receptor-deficient mice (LDLR^−/−^ mice) fed a Western-style diet showed lower levels of plasma ITIH2 compared to LDLR^−/−^ mice fed standard chow [40].

Further, fibronectin was identified to be the most probable protein target of seven aptamers. This protein is the most abundant glycoprotein in the extracellular matrix of the liver, and protein expression increases with liver injury [41]. These aptamers were capable of discriminating MASLD without steatohepatitis from MASH and F0 from F3–4. In the literature, fibronectin has been shown to play an important role in hepatic fibrogenesis [42,43] and exert modulatory functions to prevent excessive fibrosis deposition [44,45]. In humans, hepatic fibronectin is a predictor of fibrosis progression in obese individuals with MASH [46], and plasma fibronectin was used in a non-invasive score to predict fibrosis stage in individuals with chronic hepatitis C virus (HCV) [47].

Five aptamers had CFH as the most probable target. This protein belongs to the complement system and modulates the activity of the classical and alternative pathways by inhibiting the activation of complement factor C3 [48]. Although there is no direct association between CFH and human MASLD in the literature, plasma CFH concentration is positively associated with BMI, waist circumference, triglycerides, and markers of systemic inflammation [49], and CFH knockout mice develop a phenotype similar to MASLD with hepatic steatosis, fibrosis, HCC, and elevated hepatic mRNA markers of inflammation [50]. Three aptamers had C4BP as the most probable target and were, similarly to fibronectin, able to discriminate MASLD without steatohepatitis from MASH and F0 from F3–4. C4BP is a large multimer protein of the complement system, where it primarily promotes the degradation of activated C4b and thus inhibits activation of the lectin and classical pathways [51]. C4BP has been identified as a potential marker of MASLD or metabolic dysfunction by several studies. Shafiha et al. identified C4BP as a marker of MASLD by facilitating machine learning methods on transcriptomic data [52], Wang et al. found differential C4BP expression in individuals with MASH compared to healthy controls [53], and Toye et al. found C4BP expression to increase in a high-fat diet mouse model [54]. Lastly, three aptamers had IGHG3 as the most probable target. This protein comprises the constant region of the heavy chain of IgG3. In the HDL proteome, IGHG3 was significantly altered between healthy individuals and individuals with MASLD without steatohepatitis and between individuals with MASLD without steatohepatitis and MASH [39].

Altogether, five proteins were identified by MS as putative targets of the discriminative aptamers. FN, C4BP, IGHG3, and ITIH2 have been previously described as altered on either a transcriptional or protein level in MASLD. CFH is not linked directly to human MASLD but links MASLD through common inflammatory pathways, thus implicating this protein as a potential novel biomarker for MASLD. Altogether, these findings highlight the ability of APTASHAPE to identify biologically relevant proteins with the potential to function as biomarkers in MASLD. Due to limitations in the study design, we are unable to conclude on any causality between the identified proteins and MASLD.

## 6. Conclusions

In summary, we have shown that the APTASHAPE technology is a potential tool to select panels for staging MASLD disease severity. Equally important, the probable protein targets of the identified discriminative aptamers are associated with MASLD, highlighting the potential of APTASHAPE to discover novel protein biomarkers in MASLD.

## Figures and Tables

**Table 1:** Baseline characteristics of the three cohorts. Data are presented as medians (interquartile range) or n (%). * Baseline plasma samples from three individuals were unavailable. ** Histological diagnosis of MASH relied on the fatty liver inhibition of progression (FLIP) algorithm in the development and test cohorts and an NAFLD activity score (NAS) ≥4 in the bariatric cohort. Abbreviations: ALT, alanine aminotransferase; AST, aspartate aminotransferase; BMI, body mass index; FIB-4, Fibrosis-4 index; HDL, high-density lipoprotein; LDL, low-density lipoprotein; MASH, metabolic dysfunction-associated steatohepatitis; MASLD, metabolic dysfunction associated steatotic liver disease; NAS, NAFLD activity score; sCD163, soluble CD163; T2D, type 2 diabetes; N/A, not applicable.

Cohort	Development Cohort	Test Cohort		Bariatric Cohort	
Subset of samples		MASLD	Healthy	Baseline samples	EOS samples
Samples	77	47	10	59 *	62
Sex (male)	58 (75%)	31 (66%)	5 (50%)	20 (34%)	21 (34%)
Age (years)	42 (34–51)	52 (34–62	56 (52–62)	50 (36–56)	51 (37–58)
BMI (kg/m^2^)	28.3 (25.0–31.0)	34 (30.7–35.8)	32.6 (30.3–33.6)	42.0 (39.5–46,7)	38.7 (31.8–41.6)
No MASLD/MASLD Without steatohepatitis/MASH **	0/16/61	0/23/24	10/0/0	15/32/12	30/21/11
Fibrosis (F0/F1/F2/F3/F4)	26/11/18/16/6	22/23/2/0/0	10/0/0/0/0	16/32/7/2/2	15/27/15/3/2
NAS (0/1–4/5–8)	0/58/20	0/30/17	10/0/0	14/36/9	30/26/6
Bariatric surgery	N/A	N/A	N/A	0 (0%)	25 (40%)
T2D	15 (19%)	0	0	14 (24%)	17 (27%)
ALT (U/L)	68 (41–100)	52 (28–93)	30 (20–43)	31 (22–48)	30 (21–43)
AST (U/L)	48 (29–57)	46 (37–57)	N/A	25 (20–32)	24 (20–30)
Triglycerides (mmol/L)	1.4 (0.9–2.0)	1.5 (1.1–2.0)	1.45 (1.2–2.2)	1.42 (1.0–1.8)	1.29 (0.8–1.9)
Total cholesterol (mmol/L)	5.2 (4.2–6.1)	4.8 (4.2–5.7)	5.2 (4.4–5.7)	4.4 (3.9–5.1)	4.5 (3.5–5.3)
LDL (mmol/L)	3.5 (2.7–4.3)	2.9 (2.3–3.5)	3.2 (2.7–3.7)	3.0 (2.2–3.5)	3.0 (2.0–3.6)
HDL (mmol/L)	1.2 (1.0–1.4)	1.2 (0.9–1.4)	1.2 (1.0–1.4)	1.1 (0.9–1.3)	1.2 (1.0–1.5)
Liver stiffness (kPa)	7.2 (5.8–10)	4.9 (4.4–7.0)	4.0 (2.9–4.3)	7.8 (5.0–14.1)	7.2 (4.8–11.4)
sCD163	1.8 (1.3–2.5)	2.3 (2.0–2.7)	2.1 (1.6–2.3)	N/A	N/A
FIB-4	0.93 (0.65–1.22)	0.67 (0.46–1.04)	N/A	0.75 (0.51–1.17)	0.81 (0.55–1.03)
AST/ALT	0.63 (0.49–0.79)	0.51 (0.41–0.62)	N/A	0.79 (0.61–1.11)	0.81 (0.68–1.00)
CK18	190 (120–183)	N/A	N/A	N/A	N/A

**Table 2:** AUC values with 95% CIs for the selected aptamer panel to distinguish between MASLD without steatohepatitis and MASH in the development, test, and bariatric cohorts. * The development cohort did not contain any samples from healthy individuals. Abbreviations: CI, confidence intervals; EOS, end-of-study; N/A, not applicable; MASH, metabolic dysfunction-associated steatohepatitis; MASLD, metabolic dysfunction-associated steatotic liver disease.

	Healthy vs. MASLD Without Steatohepatitis	MASLD Without Steatohepatitis vs. MASH
Development cohort	N/A*	0.83 (0.72–0.94)
Test cohort	0.72 (0.54–0.90)	0.52 (0.35–0.69)
Bariatric cohort, Baseline samples	0.60 (0.41–0.79)	0.47 (0.28–0.67)
Bariatric cohort, EOS samples	0.80 (0.69–0.92)	0.49 (0.24–0.75)

**Table 3:** AUC values with 95% CIs for the selected fibrosis aptamer panels in the development, test, and bariatric cohorts. Abbreviations: CI, confidence interval; EOS, end-of-study; N/A, not applicable.

	F0 vs. F1–F2	F1–2 vs. F3–F4
Development cohort	0.60 (0.44–0.75)	0.52 (0.35–0.70)
Test cohort	0.54 (0.38–0.70)	N/A
Bariatric cohort, Baseline samples	0.69 (0.52–0.86)	0.44 (0.24–0.66)
Bariatric cohort, EOS samples	0.52 (0.34–0.70)	0.49 (0.10–0.88)

**Table 4:** An overview of the aptamers selected for target identification, the discriminative ability used to identify aptamers for protein target analysis, and the protein target identified by mass spectrometry. * Aptamers included in the aptamer panel to distinguish MASLD without steatohepatitis and MASH. # Aptamers included in the aptamer panel to distinguish F0 and F3–4. Due to the native conditions, most aptamers yielded more than one protein. Here, we define the protein with the highest intensity score as the most probable target protein. Abbreviations: LDL, low-density lipoprotein; MASH, metabolic dysfunction-associated steatohepatitis; MASLD, metabolic dysfunction-associated steatotic liver disease.

Aptamer	Discriminative Ability	Probable Target
1	LDL (<3.5 vs. ≥3.5 mmol/L)	Immunoglobulin heavy constant gamma 3
2	LDL (<3.5 vs. ≥3.5 mmol/L)	Complement factor H
3	LDL (<3.5 vs. ≥3.5 mmol/L)	Complement factor H
4	sCD163 (<1.8 mmol/L vs. ≥1.8 mmol/L)	Fibronectin/C4b-binding protein alpha chain
5	sCD163 (<1.8 mmol/L vs. ≥1.8 mmol/L)	Inter-alpha-trypsin inhibitor heavy chain H2
6	sCD163 (<1.8 mmol/L vs. ≥1.8 mmol/L)	Inter-alpha-trypsin inhibitor heavy chain H2
7	sCD163 (<1.8 mmol/L vs. ≥1.8 mmol/L)	Fibronectin
8 *#	sCD163 (<1.8 mmol/L vs. ≥1.8 mmol/L)	Fibronectin
9	sCD163 (<1.8 mmol/L vs. ≥1.8 mmol/L)	Fibronectin
10 *	sCD163 (<1.8 mmol/L vs. ≥1.8 mmol/L)	Inter-alpha-trypsin inhibitor heavy chain H2
11	sCD163 (<1.8 mmol/L vs. ≥1.8 mmol/L)	Complement factor H
12	sCD163 (<1.8 mmol/L vs. ≥1.8 mmol/L)	Complement factor H
13 *	sCD163 (<1.8 mmol/L vs. ≥1.8 mmol/L)	Inter-alpha-trypsin inhibitor heavy chain H2
14 #	sCD163 (<1.8 mmol/L vs. ≥1.8 mmol/L)	Fibronectin/C4b-binding protein alpha chain
15	sCD163 (<1.8 mmol/L vs. ≥1.8 mmol/L)	Inter-alpha-trypsin inhibitor heavy chain H2
16	sCD163 (<1.8 mmol/L vs. ≥1.8 mmol/L)	Inter-alpha-trypsin inhibitor heavy chain H2
17 #	sCD163 (<1.8 mmol/L vs. ≥1.8 mmol/L)	C4b-binding protein alpha chain
18	sCD163 (<1.8 mmol/L vs. ≥1.8 mmol/L)	C4b-binding protein alpha chain
19	Fibrosis (F1–2 vs. F3–4)	Complement factor H
20	Fibrosis (F1–2 vs. F3–4)	Immunoglobulin heavy constant gamma 3
21	Fibrosis (F1–2 vs. F3–4)	Immunoglobulin heavy constant gamma 3
22	MASLD without steatohepatitis vs. MASH	Fibronectin
23	MASLD without steatohepatitis vs. MASH	Fibronectin
24	MASLD without steatohepatitis vs. MASH	Fibronectin
25	MASLD without steatohepatitis vs. MASH	Fibronectin
26 *#	MASLD without steatohepatitis vs. MASH	C4b-binding protein alpha chain
27	Metabolic syndrome	Inter-alpha-trypsin inhibitor heavy chain H2
28 *	Metabolic syndrome	Inter-alpha-trypsin inhibitor heavy chain H2

## Data Availability

All requests for primary data should be addressed to the authors of the works contributing with data to this study.

## Supplementary Materials

The following supporting information can be downloaded at https://www.mdpi.com/article/10.3390/biom15020255/s1, Supplementary S1: Figure S1. Plot showing the count of zeros for the unique aptamers across all samples and the rank of all aptamers, i.e., most abundant aptamer rank 1, in the development cohort. Supplementary S2: Methods for affinity purification of proteins, mass spectrometry experimental setup, and mass-spectrometry data analysis.
