# Supplementary material for: Diagnosis and Staging of Metabolic Dysfunction-Associated Steatotic Liver Disease Using Biomarker-Directed Aptamer Panels
Source: Biomolecules. 2025 Feb 10;15(2):255. doi: 10.3390/biom15020255 (PMC11852711; doi:10.3390/biom15020255)
Supplement: Supplementary file 1 [file biomolecules-15-00255-s001.zip › biomolecules-3400777-supplementary.pdf]

Supplementary S1

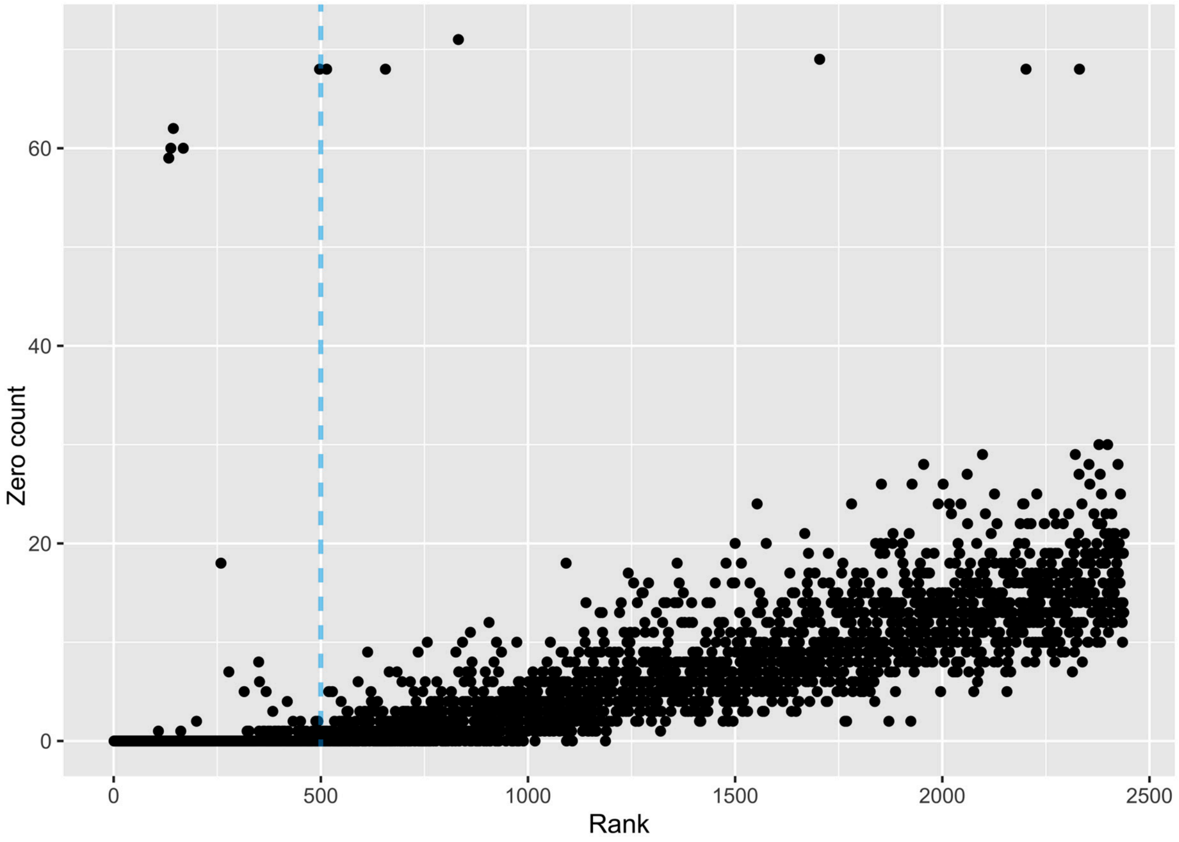

Supplementary Figure S1: Plot showing the count of zeros for the unique aptamers across all samples and the rank of all aptamers, i.e., most abundant aptamer rank 1, in the *Development cohort*. The dotted blue line indicates the threshold to include aptamers for aptamer panel development. Only the 500 most abundant aptamers in the *Development cohort* were included to increase the signal-to-noise ratio.

Supplementary S2

Affinity purification of plasma proteins

Plasma proteins were affinity purified using 3'biotinylated aptamers immobilized on streptavidin-coated magnetic beads incubated with human plasma obtained by mixing samples from the *Development cohort* representative of the histological characteristics of interest. Aptamers (1 nmole RNA) were biotinylated in 1x reaction buffer (50 mM $Tris - HCl$ pH 7.5, 10 mM $MgCl_2$, 10 mM DTT, 1.5 mM ATP, 50% PEG8000), 50 units T4 RNA ligase (Thermo Fischer Scientific™ cat. no. EL0021), 6 μM final pCp-biotin (Jena Bioscience cat.no. NU-1706-BIO), and 0.5 units inorganic pyrophosphatase (Thermo Scientific^{TM} cat.no. EF0221). The reaction was incubated for 16 hours at 4$°C$. According to manufacturer protocol, aptamers were purified using RNA Clean & Concentrator 25 spin columns (Zymo Research cat.no. R1017). Streptavidin-coated magnetic beads (Invitrogen™ cat. no. 11205D) were prepared for coupling of biotinylated RNA by washing 2 times in 200 µl 1x SELEX buffer. Biotinylated RNA was refolded by heating to 95°C and cooled to 20°C. Biotinylated RNA (400 pmole) was immobilized on 1 mg streptavidin magnetic beads (Invitrogen™ cat. no. 11205D) in 200 ul 1x HBS buffer (150 mM NaCl, 20 mM HEPES, pH 7.4) for 30 minutes. Beads were washed 2 times in 200 μl 1x HBS buffer supplemented with 1% tRNA, followed by incubation in 1x HBS buffer supplemented with 10 % plasma for 30 minutes on an orbital shaker at 900 rpm. All samples were washed 2 times in 200 μl 1x HBS buffer supplemented with 0,1% Tween20 followed by 2 wash steps in 200 µl 1x HBS buffer. Samples were moved to new tubes after each washing step. Bound proteins were eluted in 40 ul 1x HBS buffer supplemented with 20 mM EDTA and incubated for 10 minutes at 50$°C$ shaking.

Mass spectrometry

Sample preparation

Forty microliters of protein solution was reduced and alkylated by adding 40ul 6M guanidine hydrochloride (Gua), 5mM tris(2-carboxyethyl)phosphine (TCEP), 10mM chloroacetic acid (CAA), 10mM Tris buffer at pH 7.5 and heat treating for 10min at 90°C. Samples were then sonicated (Fischer Scientific) 5 x 30s on ice using cup horn indirect sonication (Qsonica). Protein concentration was measured using a NanoDrop One (Thermo

Scientific) and the protein solution was digested with Trypsin/Lys-C mixture (Promega) in an enzyme/protein ratio of 1:100 (w/w) for 16h after dilution to 1M Guanidine in 100mM TEAB.
Protease activity was quenched by acidification with trifluoroacetic acid (TFA) to a final concentration of 1%.
Peptides were cleaned and concentrated on a Sep-Pak 50mg C18 cartridge (Waters) and eluted off Sep-Pak column in 400ul steps at 40% and 60% acetonitrile (ACN). The ACN was removed from the eluate and the total volume was reduced by vacuum centrifugation at 45°C. The final concentration of the peptides was determined by 280nm absorbance on a NanoDrop One (Thermo Scientific).

Nanoflow LC-MS/MS

Volume was adjusted to 20ul in loading buffer (2% ACN, 0.1% FA, 0.1% TFA) prior to injection. An in-house packed 30cm, 75um ID capillary column with 1.9um Reprosil-Pur C18 beads (Dr. Maisch, Germany) was used. An EASY-nLC 1200 system (ThermoFisher Scientific) was used and the column temperature was maintained at 60 °C using a butterfly column oven (Phoenix S&T). Formic acid (FA) 0.1% was used as Buffer A and 80% ACN, 0.1% FA as Buffer B with a total gradient time of 60min. Gradient steps were 10% to 30%B ACN in 45min, 30% to 45%B in 5min, 45% to 80%B in 2min, 3min at 80%B followed by 80% to 5%B in 2min and a final 3min equilibration step at 5%B. Flow rate was kept at 250 nL/min.
Ionized spray was introduced to Orbitrap Exploris-480 (Thermo Scientific) using a Nanospray Flex source (Thermo Scientific)
Spray voltage was set to 2.2 kV, the heated capillary at 275 °C and RF Lens at 40%. The data was obtained in DDA mode where full MS resolution was 60,000 at m/z 200 and full MS AGC target was 3E6 with an IT of up to 25 ms. The mass range for MS/MS spectra was set to 350–1400 and AGC target value for fragment spectra was set at 1E5 with a resolution of 15,000 and injection times of 22 ms. Up to 15 fragment scans were obtained between full MS scans. The intensity threshold was kept at 2E5. Isolation width was set at 2m/z and a fixed first mass of 110m/z was used. Normalized CE was set at 30%. All data were acquired in profile mode using positive polarity.

Raw data processing and MS data analysis

DDA files were processed using MaxQuant (1.6.10.43) with default settings including Carbamidomethyl (C) as fixed modifications and Oxidation (M) and Acetyl (Protein N-term) as variable modifications. SwissProt FASTA file for humans was downloaded from Uniprot on 1st of December 2017.

The data were filtered prior to analysis by excluding samples labeled as "Reverse", "Only identified by site", and "Potential contaminant" by the Proteome Discoverer™ software using default settings. The percentage of total signal intensity was calculated for each identified protein and in each sample. The mean was calculated for each triplicate, and the mean percentage was log2 transformed. Highly enriched protein targets were identified by data visualization using hierarchical cluster analysis using the R package pheatmap.
